# Acknowledgement of *Cilia’s* reviewers in 2013

**DOI:** 10.1186/2046-2530-3-1

**Published:** 2014-01-23

**Authors:** Phil Beales, Peter K Jackson

**Affiliations:** 1 UCL Institute of Child Health, London, WC1N 1EH, UK; 2 Stanford University School of Medicine, Stanford, CA 94305-5101, USA

## Abstract

**Contributing reviewers:**

We would like to thank the following experts for their assistance with the peer-review in 2013 for manuscripts submitted to *Cilia*. We greatly appreciate the voluntary contribution made to the journal by all our reviewers.

## 

Jens Andersen

Denmark

Jose Badano

Uruguay

Alexandre Benmerah

France

Oliver Blacque

Ireland

Robert Bloodgood

United States of America

Martin Blum

Germany

Jason Brown

United States of America

Tamara Caspary

United States of America

Mark Chilvers

Canada

Soren Christensen

Denmark

Jean Cohen

France

Douglas Cole

United States of America

Pamela Cowin

United States of America

Helen Dawe

United Kingdom

James Deane

Australia

Bill Dentler

United States of America

Iain Drummond

United States of America

Benedicte Durand

France

Rachel Giles

Netherlands

Eric Haarman

Netherlands

Paul Hamel

Canada

Courtney Haycraft

United States of America

Yuqing Hou

United States of America

Jinghua Hu

United States of America

Claire Jackson

United Kingdom

Gert Jansen

Netherlands

Karl Lechtreck

United States of America

Paul Lefebvre

United States of America

Amanda Leightner

United States of America

Jarema Malicki

United Kingdom

Jeffrey Martens

United States of America

Brian Mitchell

United States of America

David Mitchell

United States of America

Hannah Mitchison

United Kingdom

Kim G Nielsen

Denmark

Heymut Omran

Germany

Greg Pazour

United States of America

Lotte Pedersen

Denmark

Elena Pugacheva

United States of America

John Sayer

United Kingdom

Jonathan Scholey

United States of America

Diane Slusarski

United States of America

Elizabeth Smith

United States of America

Tim Stearns

United States of America

Kerry Tucker

Germany

Sue Vaughan

United Kingdom

Christopher Westlake

United States of America

Mark Winey

United States of America

Bradley Yoder

United States of America

Zhibing Zhang

United States of America

